# Draft Genome Sequence of a Mycobacterium Strain Isolated from a Clinical Wound Sample

**DOI:** 10.1128/mra.00170-22

**Published:** 2022-06-02

**Authors:** Eric M. Ransom, Sanjam S. Sawhney, Gautam Dantas, Carey-Ann D. Burnham, Skye R. S. Fishbein

**Affiliations:** a Department of Pathology and Immunology, Washington University School of Medicine, St. Louis, Missouri, USA; b The Edison Family Center for Genome Sciences & Systems Biology, Washington University School of Medicine, St. Louis, Missouri, USA; c Department of Molecular Microbiology, Washington University School of Medicine, St. Louis, Missouri, USA; d Department of Biomedical Engineering, Washington University in St. Louis, St. Louis, Missouri, USA; e Departments of Pediatrics and Medicine, Washington University School of Medicine, St. Louis, Missouri, USA; University of Maryland School of Medicine

## Abstract

We report the draft genome sequence of an unusual Mycobacterium isolate recovered from a patient’s arm tissue. The 4,025,753-bp draft genome exhibits a GC content of 71.02%, and a 16S rRNA gene analysis found that the closest relative was Mycobacterium grossiae.

## ANNOUNCEMENT

Mycobacteria are aerobic, acid-fast, nonmotile, non-spore-forming bacilli, and some species, such as Mycobacterium tuberculosis, are important human pathogens. *Mycobacterium* other than tuberculosis (MOTTs), also called nontuberculous mycobacteria (NTMs), can also cause human disease. Recent changes to mycobacterial taxonomy include the subgenera *Mycolicibacterium*, *Mycolicibacter*, *Mycolicibacillus*, and *Mycobacteroides* (1); these contain over 180 species and are rapidly expanding.

A Mycobacterium-like isolate was recovered from arm tissue collected during surgical amputation following a motor vehicle accident. The isolate was detected after a 38-day incubation at 35°C in a Mycobacteria Growth Indicator Tube (MGIT) (Becton Dickinson). Clinical identification methods, including Bruker Biotyper and Vitek MS matrix-assisted laser desorption ionization–time of flight mass spectrometry (MALDI-TOF MS) systems, failed to provide a genus- or species-level identification (2). However, the isolate was acid fast and a scotochromogen (3). Subcultured growth on Middlebrook 7H10 agar in atmospheric air was observed at 30°C and 35°C after 3 days (rapid grower). The isolate was forwarded to the University of Texas at Tyler mycobacteriology laboratory for susceptibility testing and was pansusceptible at the lowest dilutions tested (Table 1) (4).

**TABLE 1:** Antimicrobial susceptibility results for mycobacterial isolate

Antimicrobial agent	MIC (μg/mL)	CLSI interpretation^*a*^
Clarithromycin	≤0.06	S
Rifabutin	≤0.25	S
Moxifloxacin	≤0.12	S
Rifampin	≤0.25	S
Trimethoprim-sulfamethoxazole	≤0.12/2.38	S
Amikacin	≤1	S
Linezolid	≤1	S
Ciprofloxacin	≤0.12	S
Doxycycline	≤0.12	S
Bedaquiline	≤0.001	S
Clofazimine	≤0.008	S

a S, susceptible. Susceptibility testing and interpretative categories were according to CLSI document M24 (4).

To characterize this isolate, it was cultured for whole-genome sequencing on Middlebrook 7H10 mycobacterial agar for 14 days at 30°C in O_2_. Colony growth was suspended in 1 mL of molecular biology-grade water. DNA was purified using the QIAamp BiOstic bacteremia DNA kit (Qiagen), followed by use of the Nextera XT library preparation kit (Illumina), and was sequenced using the NovaSeq 6000 sequencing system to acquire 2 × 150-bp paired-end reads. For all software used subsequently, default parameters were used unless otherwise specified. We generated 8,960,765 reads after quality filtering using Trimmomatic v0.36 (5). SPAdes v3.13.0 (6) was used for *de novo* assembly of a draft genome, and assembly quality was measured by QUAST (7). The draft genome was 4,025,753 bp, with a GC content of 71.02%, and consisted of 23 contigs, with an *N*_50_ value of 401,536 bp and coverage of 333×. Contigs of ≤500 bp were removed before assembly deposition. The genome was annotated with PGAP (8) and contained 3,946 coding sequences.

**FIG 1:**
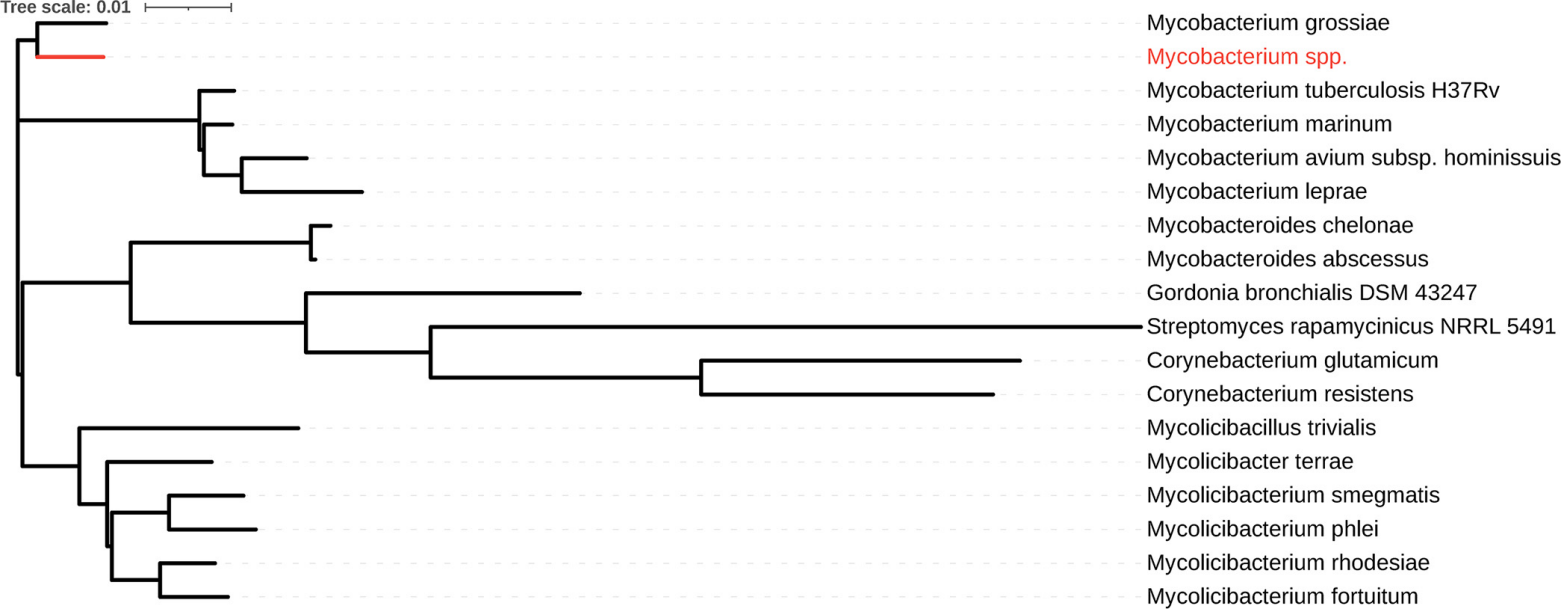
Phylogenetic tree based on 16S rRNA gene alignment of representative species (*n* = 17) from closely related *Actinobacterium* genera outside *Mycobacterium* and from each mycobacterial subgenus. The red text and node indicate the location of the isolate presented. The alignment was constructed using MUSCLE, and the approximate maximum likelihood tree was constructed with FastTree using the generalized time-reversible model. Default parameters were used for FastTree and MUSCLE. The following genomes served as sources for 16S rRNA gene sequences used in the alignment and tree: Mycobacterium leprae MRHRU-235-G (GenBank accession number NZ_CP029543.1), Mycolicibacterium smegmatis NCTC8159 (GenBank accession number NZ_LN831039.1), Mycolicibacterium rhodesiae DSM 44223 (GenBank accession number NZ_MVIH01000038.1), Mycobacterium grossiae DSM 104744 (GenBank accession number NZ_CP043474.1), Mycolicibacterium phlei NCTC8156 (GenBank accession number NZ_UGQI01000001.1), Mycolicibacter terrae NCTC10856 (GenBank accession number NZ_LT906469.1), Mycobacterium marinum MMA1 (GenBank accession number NZ_CP058277.1), Streptomyces rapamycinicus NRRL 5491 (GenBank accession number NZ_QYCY01000001.1), Mycolicibacillus trivialis DSM 44153 (GenBank accession number NZ_LQPZ01000055.1), Corynebacterium glutamicum T6-13 N_25 (GenBank accession number NZ_LOQW01000011.1), Mycobacterium avium subsp. *hominissuis* OCU464 (GenBank accession number NZ_CP009360.4), Mycobacteroides chelonae CCUG 47445 (GenBank accession number NZ_CP007220.1), Mycobacteroides abscessus FLAC013 (GenBank accession number NZ_CP014955.1), Mycolicibacterium fortuitum CT6 (GenBank accession number NZ_CP011269.1), Corynebacterium resistens DSM 45100 (GenBank accession number NC_015673.1), Gordonia bronchialis DSM 43247 (GenBank accession number NC_013441.1), and Mycobacterium tuberculosis H37Rv (GenBank accession number KK339370.1).

Using RNAmmer v1.2 (9), we isolated 16S rRNA gene sequences from the draft genome and 17 other actinomycete taxa for comparison, with pairwise identity determined using the EZBioCloud database (10). This analysis confirmed that the closest relative was Mycobacterium grossiae, with other species of the subgenus *Mycolicibacterium* having >98% identity. We aligned the corresponding gene sequences using MUSCLE and constructed an approximate maximum likelihood tree using FastTree (11, 12). Phylogenetic analysis of 16S rRNA gene alignment revealed that this Mycobacterium genomospecies and M. grossiae formed a clade distinct from other well-known representative species of the four mycobacterial subgenera (Fig. 1). Previous phylogenetic analyses of NTMs clarified that M. grossiae grouped with the fast-growing and primarily environmental mycobacterial subgenus *Mycolicibacterium* (1), although M. grossiae remains unclassified by subgenus. Given the phenotypic characteristics of this Mycobacterium genomospecies and its genotypic attributes, it may represent a missing link between the Mycobacterium and *Mycolicibacterium* subgenera.

This study was performed with institutional review board (IRB) approval from Washington University in St. Louis (IRB approval number 202204102).

### Data availability.

This whole-genome shotgun project for Mycobacterium sp. strain MYCO198283 has been deposited in GenBank under DDBJ/ENA/GenBank accession number JAJQJI000000000, BioProject accession number PRJNA759261, BioSample accession number SAMN21161762, and SRA accession number SRS11245207.
